# Potential utility of urinary chemokine CCL2 to creatinine ratio in prognosis of 5‐year graft failure and mortality post 1‐year protocol biopsy in kidney transplant recipients

**DOI:** 10.1002/iid3.901

**Published:** 2023-06-20

**Authors:** Michal Gniewkiewicz, Jolanta Gozdowska, Dominika Deborska‐Materkowska, Katarzyna Czerwinska, Agnieszka Perkowska‐Ptasinska, Anna Burban, Aleksandra Cieslik, Maciej Kosieradzki, Magdalena Durlik

**Affiliations:** ^1^ Department of Transplantation Medicine, Nephrology and Internal Medicine Medical University of Warsaw Warsaw Poland; ^2^ Department of Pathology Medical University of Warsaw Warsaw Poland; ^3^ Department of General and Transplantation Surgery Medical University of Warsaw Warsaw Poland

**Keywords:** CCL2, chemokines, kidney transplant

## Abstract

**Background:**

Chemokines (chemotactic cytokines) are small proteins which are engaged in many pathophysiological processes, including inflammation and homeostasis. In recent years, application of chemokines in transplant medicine was intensively studied. The aim of this study was to determine the utility of urinary chemokines CCL2 (C‐C motif ligand 2) and CXCL10 (C‐X‐C motif chemokine ligand 10) in prognosis of 5‐year graft failure and mortality post 1‐year protocol biopsy in renal transplant recipients.

**Methods:**

Forty patients who had a protocol biopsy 1 year after renal transplantation were included. Concentrations of CCL2 and CXCL10 in urine with reference to urine creatinine were measured. All patients were under the supervision of one transplant center. Long‐term outcomes within 5 years after 1‐year posttransplant biopsy were analyzed.

**Results:**

Urinary CCL2:Cr at the time of biopsy was significantly increased in patients who died or had graft failure. CCL2:Cr was proven to be a significant predictor of 5‐year graft failure and mortality (odds ratio [OR]: 1.09, 95% confidence interval [CI]: 1.02–1.19, *p* = .02; OR: 1.08, 95% CI: 1.02–1.16, *p* = .04; respectively).

**Conclusion:**

Chemokines are easily detected by current methods. In the era of personalized medicine, urinary CCL2:Cr can be considered as a factor providing complementary information regarding risk of graft failure or increased mortality.

## Graphical abstract

Chemokines are easily, noninvasively detected in urine by enzyme‐linked immunosorbent assay, which could facilitate their application in clinical practice. In the era of personalized medicine, urinary CCL2:Cr can be considered as a factor providing complementary information regarding the risk of graft failure or increased mortality.

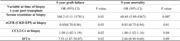

## 1 INTRODUCTION

Chemokines (chemotactic cytokines) are heparin‐binding proteins that have sizes between 5 and 20 kDa. They are engaged in many pathophysiological processes, including inflammation and homeostasis.^1^ Chemokines can bind glycosaminoglycans located on the surface of endothelial cells and in the extracellular matrix, including heparan sulfate.^2^ Moreover, chemokines interact with several receptors and most of the chemokines' receptors can be recognized by different ligands. Those receptors belong to seven transmembrane G‐protein coupled receptors, which are localized predominantly on the surface of leukocytes.^3^ Glycosaminoglycans—chemokines—receptors interactions are intensively studied.^4^ Chemokines are able to bind heparan sulfate which is a glycosaminoglycan widely expressed on cell surfaces and in the extracellular matrix, but also it can be derived from heparin—anticoagulation drug.^5^ Anti‐inflammatory effect of heparin is under investigation.^6^ Chemokines are engaged in mediating graft injury during different stages of transplantation, which reflects inflammatory/innate and donor‐specific/acquired immune responses. The production of chemokines is noted early during the reperfusion of graft, during episodes of acute rejection and during the development of vasculopathy and fibrosis.^7^, ^8^

One of the first discovered chemokine is CCL2 (C‐C motif ligand 2), also called monocyte chemoattractant protein‐1 (MCP‐1).^9^ CCL2 plays an important role in many inflammatory diseases and is involved in the development of renal diseases. CCL2 is found mainly in endothelial cells, fibroblasts, and mononuclear cells. The gene for CCL2 is situated on the q‐arm of chromosome 17.^10^ Expression of CCL2 is regulated by various molecules. Interleukin 1, tumor necrosis factor alpha, and interferon gamma are the main inducers of CCL2 expression. The inhibition of CCL2 expression is caused by anti‐inflammatory agents as glucocorticosteroids, estrogen, and retinoic acid.^11^, ^12^, ^13^ CCL2 binds mainly to CC chemokine receptor 2 (CCR2) which triggers cells such as monocytes and other immune cells that stimulate inflammation. It is a major factor driving leukocyte infiltration. CCL2 contributes to fibrosis by influencing macrophages.^14^ The role of CCL2 was described in numerous kidney diseases, such as IgA nephropathy, membranous nephropathy, glomerulosclerosis, autosomal dominant polycystic kidney disease, lupus nephritis, GBM‐induced nephritis, ANCA‐associated renal vasculitis, and diabetic nephropathy.^15^ CCL2 can be easily detected in urine. Therefore, its use as a biomarker of kidney disease has been broadly analyzed.^16^ Moreover, it was shown that patients after kidney transplantation (KTx) diagnosed with acute or chronic rejection have higher concentrations of CCL2 in urine and serum.^17^ During the rejection process, CCL2 produced in response to inflammatory stimulation, is actively involved in the recruitment of macrophages into the graft. The damaging impact of macrophages in allograft rejection is still being explored.^18^

Similarly to CCL2, chemokine CXCL10 (C‐X‐C motif chemokine ligand 10) has a role in the pathogenesis of kidney diseases such as lupus nephritis or kidney allograft dysfunction.^19^ CXCL10 is also classified as interferon gamma‐induced protein 10 (IP‐10). CXCL10 secretion is stimulated by interferon gamma, which implies its role in the development of inflammation and fibrosis.^20^ It binds principally to the CXCR3 receptor which is located mainly on activated T lymphocytes, and also on B‐cells and natural killer cells, which results in their mobilization into allograft and intensification of immune reaction.^21^ CXL10 has been proved to be associated with T‐cell‐mediated rejection and antibody‐mediated rejection.^22^, ^23^ Currently, a multicenter randomized controlled trial protocol of urine CXCL10 monitoring strategy in kidney transplant recipients is being performed.^24^

KTx is the treatment of choice for patients with end‐stage kidney disease. However, there is a worldwide shortage of organ donors and new patients are added to the waiting list for renal transplant every day.^25^ Currently, the median graft survival for deceased donor transplants is estimated to be 11.7 years.^26^

Many risk factors of kidney graft failure are determined: those linked to the donor, to surgical procedures and to recipients.^27^, ^28^ Antibody‐mediated rejection is recognized as the major cause of renal allograft failure.^29^ Early identification of the risk factors of graft failure or mortality could influence clinical decisions and be crucial in maintaining stable graft function and in prolonging patient survival.^30^

The aim of the study was to determine the utility of urinary chemokines CCL2 and CXCL10 in prognosis of 5‐year graft failure and mortality post 1‐year protocol biopsy in renal transplant recipients.

## 2 MATERIALS AND METHODS

### 2.1 Study population

This is a noninvasive study based on previous research, which its original aim was to analyze urinary concentrations of chemokines CCL2 and CXCL10 as the biomarkers of harmful processes in renal allograft.^31^

Primarily, the single‐center study consisted of 40 patients, who underwent a protocol biopsy 1 year after a receiving kidney transplant from a brain‐dead deceased donor between the years 2015 and 2017. The ultrasound‐guided biopsy procedure was done when the patients did not have any signs of infection and did not demonstrate any complaints. All the specimens were assessed according to the Banff classification by one renal pathologist.^32^ All patients were Caucasians and received a maintenance immunosuppression which included tacrolimus, mycophenolate mofetil, and steroids.

According to the biopsy results, four groups of patients were identified: patients with interstitial fibrosis and tubular atrophy (IFTA) grade II or III (*N* = 16), BK virus (BKV) nephropathy (*N* = 4), patients with mild inflammatory lesions fulfilling the criteria for mild rejection processes or borderline lesions (*N* = 11), and kidney recipients with IF/TA grade I or without any pathological changes (*N* = 15, constituted control group).

In this investigation, the patients' long‐term outcomes 5 years after the 1‐year posttransplant biopsy were assessed. During the follow‐up, all patients were under the supervision of our transplant center. The data were collected 5 years postbiopsy within 3 months range when patients did not present no signs of infection, nor exacerbation of chronic disease. Graft failure was defined as the need for permanent dialysis or retransplantation. Mortality from all causes was considered, including patients with failed graft.

### 2.2 Chemokine analysis

The analysis of urinary concentrations of chemokines was done according to protocol described previously.^31^ To correct for dilution of the urine, the concentrations of CCL2 and CXCL10 were expressed with reference to urinary creatinine.^33^ At the time of biopsy, urine samples were collected and stored for further analysis. An enzyme‐linked immunosorbent assay (ELISA) was used for a quantitative measurement of chemokines (R&D Systems). For each patient, samples were tested twice, and the mean value was used for analysis.

### 2.3 Statistical analysis

Mean values with standard deviation or medians with quartiles 1 and 3 (Q1–Q3) were used to represent categorical data, while percentages were used to represent continuous data. The Fisher exact test was used for categorical variables were compared using the Fisher exact test. Continuous variables were compared using, the two‐sample *t* test or Mann–Whitney test. The Shapiro–Wilk test was used to assess normality of distribution.

The predictive value for graft failure and patient death was analyzed using a univariate logistic regression model. Due to the relatively small sample size, multivariate regression analysis was not performed. Odds ratios (OR) with 95% confidence intervals (CI) were calculated.

*p* Value < .05 was considered statistically significant.

## 3 RESULTS

Patients' characteristics are depicted in Table 1. As it was described in a previous paper, the groups did not differ in age, sex, immunologic features (first transplantation, human leukocyte antigen [HLA] mismatch), and blood concentration of tacrolimus at the time of biopsy.^31^ Outcomes of the patients 5 years after the 1‐year posttransplant biopsy were analyzed. Regarding the immunosuppressive regimen, all patients with functioning graft still received triple immunosuppression consisting of tacrolimus, mycophenolate mofetil, and steroids. During the 5‐year follow‐up, five patients experienced graft failure and seven patients died (three patients died with functioning graft). Graft failure was caused by chronic antibody‐mediated rejection (*N* = 2), chronic allograft dysfunction of unknown etiology (*N* = 2), and recurrent urinary tract infections (*N* = 1). The causes of death were cardiovascular events (*N* = 5) or malignancy (*N* = 2). Table 2 shows a comparison of patients who had graft failure or died to other patients.

**Table 1:** Characteristics of study population.

	Research group (*N* = 25)				Control group (*N* = 15)
	Total (*N* = 25)	IFTA II–III (*N* = 16)	BKV nephropathy (*N* = 4)	Mild inflammatory lesions (*N* = 11)	
Age at biopsy, years; mean (SD)	47.68 (15.67)	46.25 (16.47)	50.50 (17.1)	45.27 (17.56)	50.15 (11.95)
*p* Value^a^	.43	.31	.89	.29	
5 years postbiopsy					
Graft failure (%)	20	31.25	0	18.18	0
*p* value^a^	.14	.04	1	.17	
Mortality (%)	28	31.25	25	27.27	0
*p* value^a^	.03	.04	.21	.06	

	**Total (*N*** = **17)**	**IFTA II‐III (*N*** = **10)**	**BKV nephropathy (*N*** = **3)**	**Mild inflammatory lesions (*N*** = **8)**	**Control group (*N*** = **15)**
Serum creatinine, mg/dL; mean (SD)	1.37 (0.34)	1.40 (0.41)	1.47 (0.21)	1.38 (0.43)	1.30 (0.22)
*p* value^a^	.48	.42	.24	.57	
Change of serum creatinine, mg/dL; mean (SD)	−0.18 (0.33)	−0.12 (0.32)	−0.48 (0.49)	−0.02 (0.24)	−0.03 (0.13)
*p* value^a^	.10	.19	.006	.55	
eGFR CKD EPI, mg/dL/1.72 m^2^; mean (SD)	58.75 (15.26)	56.67 (18.41)	55.67 (4.04)	59.14 (15.99)	58.53 (15.49)
*p* value^a^	.97	.79	.76	.93	
Change of eGFR CKD EPI, mg/dL/1.72 m^2^; mean (SD)	7.06 (11.39)	5.67 (12.51)	13.33 (12.06)	2.71 (9.46)	0.33 (7.27)
*p* value^a^	.06	.28	.02	.52	
Blood concentration of tacrolimus, ng/mL; mean (SD)	5.57 (1.21)	5.66 (1.34)	4.77 (1.36)	6.39 (0.94)	5.98 (1.27)
*p* value^a^	.62	.66	.28	.24	
Change of blood concentration of tacrolimus, ng/mL; mean (SD)	−1.12 (2.31)	−0.50 (2.51)	−2.47 (2.39)	−0.55 (2.32)	−0.93 (1.88)
*p* value^a^	.79	.62	.23	.67	

Abbreviations: BKV, BK virus; CKD EPI, chronic kidney disease epidemiology collaboration; eGFR, estimated glomerular filtration rate; HLA, human leukocyte antigen; IFTA, interstitial fibrosis and tubular atrophy; SD, standard deviation.

^a^ *p* Values compared with control group.

**Table 2:** Comparison of patients who had graft failure or died to alive patients with functioning graft.

	Alive patients with functioning graft (*N* = 32)	Patients with graft failure (*N* = 5)	Deceased patients (*N* = 7)
Age at biopsy, years; mean (SD)	48.19 (13.44)	48.00 (21.18)	55.00 (19.13)
*p* Value^a^		.98	.27
Male (%)	68.75	80.00	85.71
*p* Value^a^		.61	.37
First transplantation, %	12.5	20.00	14.29
*p* Value^a^		.65	.90
HLA mismatch			
Total; mean (SD)	3.19 (1.36)	4.00 (0.71)	4.14 (0.90)
*p* Value^a^		.21	.91
A mismatch; mean (SD)	1.12 (0.65)	1.60 (0.55)	1.43 (0.79)
*p* Value^a^		.18	.30
B mismatch; mean (SD)	1.31 (0.68)	1.60 (0.55)	1.57 (0.53)
*p* Value^a^		.45	.44
DR mismatch; mean (SD)	0.73 (0.60)	0.80 (0.84)	1.14 (0.69)
*p* Value^a^		.93	.20
Serum creatinine at biopsy, mg/dL; mean (SD)	1.44 (0.30)	2.14 (0.43)	2.13 (0.48)
*p* Value^a^		.004	.001
eGFR CKD EPI at biopsy, mg/dL/1.72 m^2^; mean (SD)	54.84 (13.06)	35.50 (7.72)	34.67 (8.98)
*p* Value^a^		.007	.001
CCL2:Cr, ng/mmol at biopsy; median (Q1–Q3)	8.45 (6.47‐14.95)	30.76 (15.35‐47.46)	24.34 (10.62‐30.86)
*p* Value^a^		.009	.005
CXCL10:Cr, ng/mmol at biopsy; median (Q1–Q3)	3.52 (1.48‐8.17)	7.85 (5.43‐9.84)	6.41 (3.45‐7.85)
*p* Value^a^		.14	.25
Blood concentration of tacrolimus at biopsy, ng/mL; mean (SD)	6.73 (2.06)	5.94 (1.75)	6.85 (1.62)
*p* Value^a^		.41	.90
IFTA stage			
0 (%)	18.75	0	0
1 (%)	50.00	0	28.57
2 (%)	25.00	60.00	57.14
3 (%)	6.25	40.00	14.29
*p* Value^a^		.01	.30

Abbreviations: CCL2, C‐C motif ligand 2; CKD EPI, chronic kidney disease epidemiology collaboration; CXCL10, C‐X‐C motif chemokine ligand 10; eGFR, estimated glomerular filtration rate; HLA, human leukocyte antigen; IFTA, interstitial fibrosis and tubular atrophy; SD, standard deviation.

^a^ *p* Values compared with alive patients with functioning graft.

Alive patients with functioning graft had significantly lower serum creatinine concentration at the time of biopsy and significantly higher estimated glomerular filtration rate (eGFR) Chronic Kidney Disease Epidemiology Collaboration (CKD EPI). The urinary chemokine CCL2‐to‐creatinine ratio (CCL2:Cr) at the time of biopsy was significantly increased in patients who had graft failure or died. In contrast, the urinary chemokine CXCL10‐to‐creatinine ratio (CXCL10:Cr) ratio did not differ between groups. There was significant difference regarding IFTA stage between patients with graft failure and alive patients with functioning graft.

Prognostic values of serum creatinine, eGFR CKD EPI, urinary CCL2:Cr ratio, and IFTA stage 1‐year posttransplant for graft failure or mortality are depicted in Table 3. Serum creatinine, eGFR CKD EPI, and urinary CCL2:Cr at the time of biopsy proved to be significant predictors of graft failure or mortality within 5 years. IFTA stage was identified as a significant predictor of graft failure.

**Table 3:** Logistic regression analysis model.

Variable at time of biopsy 1‐year posttransplant	5‐year graft failure		5‐year mortality	
	OR (95% CI)	*p* Value	OR (95% CI)	*p* Value
Serum creatinine at biopsy	104.2 (5.11–15781)	.01	68.69 (5.49–4367)	.007
eGFR (CKD‐EPI) at biopsy	0.85 (0.70–0.96)	.03	0.85 (0.73–0.94)	.01
CCL2:Cr at biopsy	1.09 (1.02–1.19)	.02	1.08 (1.02–1.16)	.04
IFTA	7.53 (1.87–58.07)	.02	2.66 (0.90–9.60)	.09

Abbreviations: CCL2, C‐C motif ligand 2; CI, confidence interval; CKD‐ EPI, Chronic Kidney Disease Epidemiology Collaboration; eGFR, estimated glomerular filtration rate; IFTA, interstitial fibrosis and tubular atrophy; OR, odds ratio.

## 4 DISCUSSION

Early transplant outcomes have improved over time as a result of better surgical methods, postoperative management, and modern immunosuppressives (especially calcineurin inhibitors). Long‐term results, on the other hand, have not changed to the same level, which remains challenging for transplant specialists.^34^ There is an emerging need for the ability to early detect renal allografts with a high risk of failure. Specific biomarkers with practical diagnostic and prognostic value are demanded.^35^

Urine is the most common specimen for renal diseases because it includes molecules and cell components derived precisely from glomerular filtration of plasma, excretion of kidney tubules, and secretion of the urinary tract that demonstrate pathophysiological and metabolic processes in human beings at a specific point in time. Urine samples can be obtained recurrently, in a fully noninvasive way, in reasonably large volumes.^36^, ^37^

In the study, patients with IFTA stage II or III diagnosed in biopsy 1 year after transplantation had significantly higher incidence of 5‐year graft failure or 5‐year mortality compared with patients without any pathological changes detected by biopsy. Moreover, IFTA was identified to be a predictor of graft failure. Similar results were shown in the literature.^38^, ^39^ IFTA is a feature of progressive chronic allograft dysfunction, which is a multifactorial entity and one of the main causes of long‐term graft failure. Typically, it does not give any symptoms and develops 1 year after transplantation. It results in steady decline in kidney function.^40^ The prompt diagnosis of this process is important, since failure to do so results in permanent changes and eventually to graft failure.^41^ The molecular processes engaged in chronic allograft dysfunction happen very early. However, they do not have clinical demonstration, and it is not possible to identify them by common techniques. Diagnosis of IFTA requires a renal biopsy, which is an invasive procedure.^42^

In relation to the results, it is broadly demonstrated in available papers that renal function at 1‐year posttransplant is associated with long‐term outcomes. Both serum creatinine and eGFR CKD EPI are significantly associated with graft failure and mortality.^30^, ^43^, ^44^ Nevertheless, serum creatine is affected by multiple factors including age, gender, race, body mass, diet, some of which are eliminated by glomerular filtration rate estimated equitation.^45^ Glomerular filtration rate measurement by iothalamate or other direct methods is still considered the gold standard for assessing renal function, but it would be impractical to apply this technique in routine clinical practice.^46^

Previous papers have shown that urinary concentration of CCL2 is elevated in patients with advanced IFTA or graft rejection.^31^, ^47^ Our data indicates that urinary CCL2:Cr 1‐year posttransplant is associated with 5‐year graft failure and mortality. Some publications display related findings. Increased concentration of CCL2 in kidney transplant recipients with poor outcomes may be explained by its contribution to repair after kidney injury.^48^, ^49^ CCL2 is involved in pathogenic processes during all stages of transplantation, including vasculopathy and fibrosis.^7^ CCL2 is a potent chemoattractant that regulates migration and infiltration of macrophages. Macrophage infiltration identified in 1‐year posttransplant protocol biopsies was associated with subclinical alloimmune inflammation, injury of tubules, and progression of fibrosis.^50^ In the study, mortality, could be explained by previous occurrences of graft failure (four patients out of seven died with previous graft failure). The presence of a failed renal graft after cessation of immunosuppressive treatment can lead to a state of chronic inflammation, which reduces patient survival.^51^, ^52^

There was no statistical difference regarding urinary CXCL10:Cr between patients who had graft failure or died and patients with stable graft function. It is intriguing as CXCL10 is determined as an independent predictor of long‐term graft outcome. Hirt‐Minkowski et al. confirmed in their study that urinary CCL2:Cr and CXCL10:Cr measured 6 months after KTx provide prognostic information regarding renal graft outcomes and they have similar performance. However, that study included 185 patients, of which 52 reached primary outcome at a median 6 years.^48^ Our results could be explained by smaller number of patients in the study and shorter time of observation. Moreover, Raza et al., in the study involving 273 patients, proved that urinary CXCL10 is significantly elevated at the time of renal allograft rejection.^20^

There are several limitations of the study. It is single‐center, includes relatively small sample size, and it is based on previous study. Sample size limited the application of the multivariable model. Another limitation is that concentrations of urinary chemokines were measured only 1‐year after transplant at the time of biopsy. One more concern is that all biopsy specimens were assessed by one pathologist. Moreover, mortality from all causes was considered in all patients, including patients with failed graft, in which dialysis could increase the risk of death.

## 5 CONCLUSIONS

Chemokines are easily, noninvasively detected in urine by ELISA, which could facilitate their application in clinical practice. In the era of personalized medicine, urinary CCL2:Cr can be considered as a factor providing complementary information regarding the risk of graft failure or increased mortality.

It could influence individualized therapeutic interventions and follow‐up strategies.

## AUTHOR CONTRIBUTIONS

**Michal Gniewkiewicz**: Conceptualization (equal); data curation (equal); formal analysis (lead); methodology (equal); project administration (lead); visualization (lead); writing—original draft (lead). **Jolanta Gozdowska**: Conceptualization (equal); methodology (equal); project administration (supporting); supervision (lead); writing—review and editing (equal). **Dominika Deborska‐Materkowska**: Resources (equal). **Katarzyna Czerwinska**: Investigation (equal); resources (equal); validation (lead). **Agnieszka Perkowska‐Ptasinska**: Investigation (equal); resources (equal). **Anna Burban**: Data curation (equal); investigation (equal). **Aleksandra Cieslik**: Data curation (equal); investigation (equal). **Maciej Kosieradzki**: Resources (equal). **Magdalena Durlik**: Project administration (supporting); writing—review and editing: (equal).

## CONFLICT OF INTEREST STATEMENT

The authors declare no conflict of interest.

## ETHICS STATEMENT

The study has approval from the Ethics Committee of the Medical University of Warsaw (KB/114/2014). Informed written consent was obtained from all patients. The study was performed according to the principles expressed in the Declaration of Helsinki.

## Data Availability

Data generated or analyzed during this study are included.

## References

[1] Abraham M , Wald H , Vaizel‐Ohayon D , et al. Development of novel promiscuous anti‐chemokine peptibodies for treating autoimmunity and inflammation. Front Immunol. 2017;8:1432. 10.3389/fimmu.2017.01432 29218043 PMC5703867

[2] Thompson S , Martínez‐Burgo B , Sepuru K , et al. Regulation of chemokine function: the roles of GAG‐binding and post‐translational nitration. Int J Mol Sci. 2017;18:1692. 28771176 10.3390/ijms18081692 PMC5578082

[3] Hughes CE , Nibbs RJB . A guide to chemokines and their receptors. FEBS J. 2018;285:2944‐2971. 10.1111/febs.14466 29637711 PMC6120486

[4] Crijns H , Vanheule V , Proost P . Targeting chemokine‐glycosaminoglycan interactions to inhibit inflammation. Front Immunol. 2020;11:483. 10.3389/fimmu.2020.00483 32296423 PMC7138053

[5] Sepuru KM , Rajarathnam K . Structural basis of chemokine interactions with heparan sulfate, chondroitin sulfate, and dermatan sulfate. J Biol Chem. 2019;294:15650‐15661. 10.1074/jbc.RA119.009879 31455633 PMC6816105

[6] Collins LE , Troeberg L . Heparan sulfate as a regulator of inflammation and immunity. J Leukoc Biol. 2019;105:81‐92. 10.1002/JLB.3RU0618-246R 30376187

[7] Schenk AD , Rosenblum JM , Fairchild RL . Chemokine‐directed strategies to attenuate allograft rejection. Clin Lab Med. 2008;28:441‐454. 10.1016/j.cll.2008.07.004 19028262 PMC2632546

[8] Crescioli C . Chemokines and transplant outcome. Clin Biochem. 2016;49:355‐362. 10.1016/j.clinbiochem.2015.07.026 26238260

[9] Haller H , Bertram A , Nadrowitz F , Menne J . Monocyte chemoattractant protein‐1 and the kidney. Curr Opin Nephrol Hypertens. 2016;25:42‐49. 10.1097/mnh.0000000000000186 26625862

[10] Deshmane SL , Kremlev S , Amini S , Sawaya BE . Monocyte chemoattractant protein‐1 (MCP‐1): an overview. J Interferon Cytokine Res. 2009;29:313‐326. 10.1089/jir.2008.0027 19441883 PMC2755091

[11] Sung FL , Zhu TY , Au‐Yeung KKW , Siow YL , Karmin O . Enhanced MCP‐1 expression during ischemia/reperfusion injury is mediated by oxidative stress and NF‐κB. Kidney Int. 2002;62:1160‐1170. 10.1111/j.1523-1755.2002.kid577.x 12234286

[12] Ruiz‐Ortega M , Lorenzo O , Egido J . Angiotensin III increases MCP‐1 and activates NF‐кB and AP‐1 in cultured mesangial and mononuclear cells. Kidney Int. 2000;57:2285‐2298. 10.1046/j.1523-1755.2000.00089.x 10844599

[13] Kim MJ , Tam FWK . Urinary monocyte chemoattractant protein‐1 in renal disease. Clin Chim Acta. 2011;412:2022‐2030. 10.1016/j.cca.2011.07.023 21851811

[14] Singh S , Anshita D , Ravichandiran V . MCP‐1: function, regulation, and involvement in disease. Int Immunopharmacol. 2021;101:107598. 10.1016/j.intimp.2021.107598 34233864 PMC8135227

[15] Tam FWK , Ong ACM . Renal monocyte chemoattractant protein‐1: an emerging universal biomarker and therapeutic target for kidney diseases? Nephrol Dial Transplant. 2020;35:198‐203. 10.1093/ndt/gfz082 31089695

[16] Puthumana J , Thiessen‐Philbrook H , Xu L , et al. Biomarkers of inflammation and repair in kidney disease progression. J Clin Invest. 2021;131:e139927. 10.1172/jci139927 33290282 PMC7843225

[17] Krüger B , Schröppel B , Ashkan R , et al. A monocyte chemoattractant protein‐1 (MCP‐1) polymorphism and outcome after renal transplantation. JASN. 2002;13:2585‐2589. 10.1097/01.asn.0000031701.53792.54 12239249

[18] Mannon RB . Macrophages: contributors to allograft dysfunction, repair, or innocent bystanders. Curr Opin Organ Transplant. 2012;17:20‐25. 10.1097/MOT.0b013e32834ee5b6 22157320 PMC3319132

[19] Gao J , Wu L , Wang S , Chen X . Role of chemokine (C‐X‐C Motif) ligand 10 (CXCL10) in renal diseases. Mediators Inflamm. 2020;2020:1‐16. 10.1155/2020/6194864 PMC7025113 32089645

[20] Raza A , Firasat S , Khaliq S , et al. The association of urinary interferon‐gamma inducible protein‐10 (IP10/CXCL10) levels with kidney allograft rejection. Inflamm Res. 2017;66:425‐432. 10.1007/s00011-017-1025-7 28246678

[21] Lo DJ , Kaplan B , Kirk AD . Biomarkers for kidney transplant rejection. Nat Rev Nephrol. 2014;10:215‐225. 10.1038/nrneph.2013.281 24445740

[22] Ho J , Rush DN , Karpinski M , et al. Validation of urinary CXCL10 as a marker of borderline, subclinical, and clinical tubulitis. Transplantation. 2011;92:878‐882. 10.1097/TP.0b013e31822d4de1 21876477

[23] Rabant M , Amrouche L , Lebreton X , et al. Urinary C‐X‐C motif chemokine 10 independently improves the noninvasive diagnosis of antibody‐mediated kidney allograft rejection. JASN. 2015;26:2840‐2851. 10.1681/asn.2014080797 25948873 PMC4625672

[24] Ho J , Sharma A , Kroeker K , et al. Multicentre randomised controlled trial protocol of urine CXCL10 monitoring strategy in kidney transplant recipients. BMJ Open. 2019;9:e024908. 10.1136/bmjopen-2018-024908 PMC6500325 30975673

[25] McCormick F , Held PJ , Chertow GM . The terrible toll of the kidney shortage. JASN. 2018;29:2775‐2776. 10.1681/asn.2018101030 30420419 PMC6287861

[26] Poggio ED , Augustine JJ , Arrigain S , Brennan DC , Schold JD . Long‐term kidney transplant graft survival‐making progress when most needed. Am J Transplant. 2021;21:2824‐2832. 10.1111/ajt.16463 33346917

[27] Wong RBK , Minkovich M , Famure O , et al. Surgical site complications in kidney transplant recipients: incidence, risk factors and outcomes in the modern era. Can J Surg. 2021;64:E669‐E676. 10.1503/cjs.015820 34933944 PMC8711553

[28] Betjes MGH , Roelen DL , van Agteren M , Kal‐van Gestel J . Causes of kidney graft failure in a cohort of recipients with a very long‐time follow‐up after transplantation. Front Med. 2022;9:842419. 10.3389/fmed.2022.842419 PMC9207199 35733857

[29] Mayer KA , Budde K , Jilma B , Doberer K , Böhmig GA . Emerging drugs for antibody‐mediated rejection after kidney transplantation: a focus on phase II & III trials. Expert Opin Emerg Drugs. 2022;27:151‐167. 10.1080/14728214.2022.2091131 35715978

[30] Stamenic D , Rousseau A , Essig M , et al. A prognostic tool for individualized prediction of graft failure risk within ten years after kidney transplantation. J Transplant. 2019;2019:1‐10. 10.1155/2019/7245142 PMC6476124 31093367

[31] Gniewkiewicz MS , Czerwińska M , Gozdowska J , et al. Urinary levels of CCL2 and CXCL10 chemokines as potential biomarkers of ongoing pathological processes in kidney allograft: an association with BK virus nephropathy. Pol Arch Med Wewn. 2019;129:592‐597. 10.20452/pamw.14926 31389404

[32] Haas M . The revised (2013) Banff classification for antibody‐mediated rejection of renal allografts: update, difficulties, and future considerations. Am J Transplant. 2016;16:1352‐1357. 10.1111/ajt.13661 26696524

[33] Tang K , Toh Q , Teo B . Normalisation of urinary biomarkers to creatinine for clinical practice and research‐‐when and why. Singapore Med J. 2015;56:7‐10. 10.11622/smedj.2015003 25640093 PMC4325562

[34] Coemans M , Süsal C , Döhler B , et al. Analyses of the short‐ and long‐term graft survival after kidney transplantation in Europe between 1986 and 2015. Kidney Int. 2018;94:964‐973. 10.1016/j.kint.2018.05.018 30049474

[35] Lepoittevin M , Kerforne T , Pellerin L , Hauet T , Thuillier R . Molecular markers of kidney transplantation outcome: current omics tools and future developments. Int J Mol Sci. 2022;23:6318. 10.3390/ijms23116318 35682996 PMC9181061

[36] Harpole M , Davis J , Espina V . Current state of the art for enhancing urine biomarker discovery. Expert Rev Proteomics. 2016;13:609‐626. 10.1080/14789450.2016.1190651 27232439 PMC4978532

[37] Yang JYC , Sarwal RD , Fervenza FC , Sarwal MM , Lafayette RA . Noninvasive urinary monitoring of progression in IgA nephropathy. Int J Mol Sci. 2019;20:4463. 10.3390/ijms20184463 31510053 PMC6770813

[38] Parajuli S , Aziz F , Garg N , et al. Histopathological characteristics and causes of kidney graft failure in the current era of immunosuppression. World J Transplant. 2019;9:123‐133. 10.5500/wjt.v9.i6.123 31750089 PMC6851501

[39] Terrec F , Noble J , Naciri‐Bennani H , et al. Protocol biopsies on de novo renal‐transplants at 3 months after surgery: impact on 5‐year transplant survival. J Clin Med. 2021;10:3635. 10.3390/jcm10163635 34441931 PMC8397165

[40] Goldberg RJ , Weng FL , Kandula P . Acute and chronic allograft dysfunction in kidney transplant recipients. Med Clin North Am. 2016;100:487‐503. 10.1016/j.mcna.2016.01.002 27095641

[41] Pascual J , Pérez‐Sáez MJ , Mir M , Crespo M . Chronic renal allograft injury: early detection, accurate diagnosis and management. Transplant Rev. 2012;26:280‐290. 10.1016/j.trre.2012.07.002 22902496

[42] Shrestha BM , Haylor J . Biological pathways and potential targets for prevention and therapy of chronic allograft nephropathy. Biomed Res Int. 2014;2014:1‐13. 10.1155/2014/482438 PMC4058292 24971332

[43] Hariharan S , McBride MA , Cherikh WS , Tolleris CB , Bresnahan BA , Johnson CP . Post‐transplant renal function in the first year predicts long‐term kidney transplant survival. Kidney Int. 2002;62:311‐318. 10.1046/j.1523-1755.2002.00424.x 12081593

[44] Lenihan CR , O'Kelly P , Mohan P , et al. MDRD‐estimated GFR at one year post‐renal transplant is a predictor of long‐term graft function. Ren Fail. 2008;30:345‐352. 10.1080/08860220801947686 18569905

[45] Levey AS , Stevens LA , Schmid CH , et al. A new equation to estimate glomerular filtration rate. Ann Intern Med. 2009;150:604‐612. 10.7326/0003-4819-150-9-200905050-00006 19414839 PMC2763564

[46] Huang Y , Tilea A , Gillespie B , et al. Understanding trends in kidney function 1 year after kidney transplant in the United States. JASN. 2017;28:2498‐2510. 10.1681/asn.2016050543 28270413 PMC5533222

[47] Raza A , Firasat S , Khaliq S , et al. Monocyte chemoattractant protein‐1 (MCP‐1/CCL2) levels and its association with renal allograft rejection. Immunol Invest. 2017;46:251‐262. 10.1080/08820139.2016.1248559 27960564

[48] Hirt‐Minkowski P , Rush DN , Gao A , et al. Six‐month urinary CCL2 and CXCL10 levels predict long‐term renal allograft outcome. Transplantation. 2016;100:1988‐1996. 10.1097/tp.0000000000001304 27548845

[49] Mockler C , Sharma A , Gibson IW , et al. The prognostic value of urinary chemokines at 6 months after pediatric kidney transplantation. Pediatr Transplant. 2018;22:e13205. 10.1111/petr.13205 29733487

[50] Toki D , Zhang W , Hor KLM , et al. The role of macrophages in the development of human renal allograft fibrosis in the first year after transplantation. Am J Transplant. 2014;14:2126‐2136. 10.1111/ajt.12803 25307039

[51] Gill JS , Abichandani R , Kausz AT , Pereira BJG . Mortality after kidney transplant failure: the impact of non‐immunologic factors. Kidney Int. 2002;62:1875‐1883. 10.1046/j.1523-1755.2002.00640.x 12371992

[52] Buturović‐Ponikvar J , Gubens̆ek J , Arnol M , Kandus A , Bren A , Ponikvar R . High mortality in the first year after kidney graft failure. Transplant Proc. 2013;45:1431‐1434. 10.1016/j.transproceed.2013.01.102 23726589

